# Long-Term Outcomes of Recipients of Liver Transplants from Living Donors Treated with a Very Low-Calorie Diet

**DOI:** 10.1155/2024/9024204

**Published:** 2024-05-02

**Authors:** Hannah Wozniak, Sara Naimimohasses, Toru Goto, Gonzalo Sapisochin, Blayne Sayed, Anand Ghanekar, Mark Cattral, Nazia Selzner

**Affiliations:** ^1^Interdepartmental Division of Critical Care Medicine, University of Toronto, Toronto, ON, Canada; ^2^Multi-Organ Transplant Program, Toronto General Hospital, University Health Network, Toronto, ON, Canada

## Abstract

The increasing prevalence of steatotic liver disease (SLD) in potential living donors is concerning, as it limits donor's availability amid rising demand. OPTIFAST very low-calorie diet (VLCD), a meal replacement product, effectively reduces weight and hepatic steatosis before transplantation. However, data on the outcomes of recipients of VLCD-treated donors are lacking. We conducted a single-center, retrospective study on 199 living donor liver transplant recipients at Toronto General Hospital, Canada, between January 2015 and January 2020. We compared the 1-year posttransplant outcomes between recipients who received organs from donors treated with VLCD (*N* = 34) for either weight loss or steatosis reduction, with those who did not require treatment (*N* = 165). Our analysis revealed no statistically significant differences in the rates of postoperative complications (23% vs 32.4%, *p*=0.3) or intensive care unit stays (70.9% vs 70.6%, *p*=1) between recipients of non-VLCD and VLCD grafts. Following adjusted multivariate logistic regression, receipt of VLCD grafts was not associated with increased hospital length of stay. In addition, one-year mortality did not differ between the two groups (4.2% non-VLCD recipients vs 2.9% VLCD recipients, *p*=0.6). OPTIFAST VLCD treatment for liver donors demonstrates positive and safe outcomes in recipients, expanding the pool of potential living donors for increased organ availability.

## 1. Introduction

The global obesity epidemic has led to a steady rise in the prevalence of steatotic liver disease (SLD) with an estimated prevalence of 32.4% worldwide, climbing to 47.8% in North America [1]. As the leading cause of liver-related morbidity and mortality, SLD not only contributes to the ever-increasing demand for liver transplantation, but also limits the availability of suitable donors, with rates as high as 20% reported amongst the potential living donor population [2]. In the context of organ shortage, where the number of patients waitlisted surpasses the number of successful transplants, it becomes crucial to seek solutions that can maximize the opportunities for liver living donation [3, 4].

Steatotic grafts are more vulnerable to ischemia/reperfusion injury and are associated with poorer posttransplant outcomes [5]. This includes more frequent rates of primary graft nonfunction, rejection, and biliary complications resulting in a significantly lower one-year graft survival [6–9]. Currently, a threshold of <30% steatosis is considered a safe and suitable cut-off for use in deceased donor liver transplantation in most programs [10]. Weight loss remains a cornerstone for the improvement and reversal of hepatic steatosis. The judicious use of effective weight loss interventions may help to address the organ shortage by increasing the pool of suitable potential living donors.

OPTIFAST is a commercially available, very low-calorie diet (VLCD) meal replacement product that has been shown to result in significant, rapid weight loss and improve hepatic steatosis [11, 12]. Since 2012, our program has used this VLCD product to achieve weight loss and/or defatting of potential donors. We previously reported the safety and efficacy of this VLCD in the living donor liver population [13]. This study now focuses on the long-term outcomes of the transplant recipients who had a graft from donors on VLCD predonation.

## 2. Materials and Methods

### 2.1. Study Design

We conducted a retrospective, single-center, observational study aimed at analyzing adult living donor liver recipients at the Ajmera Transplant Center at Toronto General Hospital in Toronto, Ontario. We recruited all OPTIFAST VLCD patients from 2012 to 2020 and compared them with non-VLCD patients who underwent LDLT between 2015 and 2020. The extension of follow-up time for the VLCD group was necessary to reach a sufficient sample size, ensuring a comprehensive and robust comparison with the non-VLCD control group.

VLCD donors were defined as donors treated with OPTIFAST VLCD predonation for hepatic steatosis (>7%), diagnosed through MRI-MRE and/or BMI of >30 kg/m^2^. The OPTIFAST VLCD is offered in our center as a standard of care to potential donors in this specific context and they may accept or decline after receiving all necessary information. The VLCD was prescribed by the transplant hepatologist (NS) and donors were closely monitored through the diet as described previously [13]. The length of treatment was determined by the hepatologist, based on the amount of weight lost, typically ranging from two to eight weeks. After completing the VLCD treatment, all donors with evidence of hepatic steatosis underwent a liver biopsy prior to proceeding with organ donation.

Recipients were divided into two groups based on whether they received a liver from a VLCD-treated donor or a non-VLCD-treated donor. For each liver recipient, demographic data, reasons for a liver transplant, and model for end-stage liver disease (MELD) score were recorded. Information pertaining to the perioperative period was also documented. In the posttransplantation period, laboratory values, data on the need for intensive care unit (ICU) admission, duration of ICU stay, hospital length of stay, complications in the first 90 days, and mortality at day 30, 90, and 360 were collected. Complications included new graft thrombosis, biliary stricture, postsurgical bleeding, infections, and small-for-size syndrome.

To assess the possible recurrence of liver steatosis at one year in non-SLD recipients who received a VLCD graft, we performed a chart review of all imaging and biopsies performed in the first year following transplantation. We assessed graft steatosis using ultrasound, CT scan, MRI, and available biopsy data, categorizing its presence as binary (present or absent) [14].

This study was approved by the Research Ethics Board of the University Health Network, Toronto, and was conducted in accordance with both the Declarations of Helsinki and Istanbul. The authors and the transplant center declare that they have not received funding from, nor hold any conflicts of interest with, the manufacturers of the VLCD OPTIFAST diet product or any of their affiliates.

### 2.2. Statistical Analysis

First, donors were categorized and described based on whether they underwent a VLCD. Second, recipients were classified into two groups based on whether they received an organ from a donor who had been treated with a VLCD or from one who had not, resulting in groups identified as VLCD recipients and non-VLCD recipients.

Categorical variables were presented as numbers (*n*) and percentages (%). Continuous variables were presented as median and interquartile range (IQR). Fisher's exact test was used to determine statistical differences in categorical variables. For continuous variables, a Mann–Whitney *U* test was conducted.

To evaluate the potential effects of VLCD treatment on recipient outcomes, we selected *a priori* hospital length of stay (LOS) as a surrogate marker for postoperative complications and resource utilization. Subsequently, we conducted a multivariate logistic regression model analysis. Prolonged hospital length of stay was defined as more than 13 days, which corresponded to the median length of stay of the entire study population. Variables in the logistic regression were defined *a priori* as possible confounding factors. Results of the logistic regression are expressed as odds ratio (OR) and 95% confidence intervals (95% CI).

A complete case analysis was performed due to the low number of missing data. Two-tailed *p* values of ≤0.05 were considered statistically significant. Statistical analyses were performed using STATA version 16.1 (StataCorp., College Station, TX, USA, 2007).

## 3. Results

### 3.1. Donor's Characteristics

Table 1 presents the characteristics of the 165 donors who did not receive VLCD (non-VLCD donors) and the 34 donors who received VLCD (VLCD donors). There were no significant differences between the two groups in terms of age or sex distribution. The median body mass index (BMI) of the non-VLCD donors upon donation was lower than the BMI of VLCD donors (25.2 (22.6–27.7) vs. 28.1 (26.5–29.7), *p* < 0.01). No significant difference in the liver enzyme tests was noted between the two groups.

### 3.2. Recipient's Characteristics and Outcomes

Table 2 presents the characteristics of the recipients of living donor grafts and compares non-VLCD recipients with VLCD recipients. In the study population, 48.2% were women (99/199). The main indications for organ transplantation included primary sclerosing cholangitis, SLD, viral hepatitis, and alcoholic liver cirrhosis. No significant differences were found between the two groups regarding the indication for transplantation or the MELD score before LT.

Non-VLCD recipients had a lower graft-to-recipient weight ratio (1.1 (1–1.4) vs 1.3 (1–1.7), *p*=0.04) but both were above the 0.8 accepted threshold [15]. Non-VLCD recipients tended to have a longer duration of surgery (557.5 minutes (480–650) vs 508.5 minutes (435–600), *p*=0.09), although the difference was not statistically significant (Table 3).

In the posttransplantation period, no differences were found in the peak of AST, ALT, and bilirubin. However, the peak of INR post-LT was higher in the non-VLCD recipients (2.2 (1.7–2.6) vs 1 (1–1.25), *p* < 0.01). The complication rate appears to be the same between the two groups, with overall 13.1% (26/199) experiencing biliary stricture and 3.5% (7/199) experiencing hepatic thrombosis. Six non-VLCD recipients had postoperative intraabdominal bleeding, and three required surgical laparotomy, whereas none of the VLCD recipients experienced these complications. There was no difference in the need for ICU stay (117 (70.9%) of non-VLCD recipients vs 24 (70.6%) VLCD recipients, *p*=1) postliver transplant. The length of hospital stay did not differ between the two groups (13 (9–22) for non-VLCD recipients vs 13 (9–24) for VLCD recipients, *p*=0.8). Mortality at day 30, 90, and 1 year did not differ between the two groups, with 4.2% (7/165) of non-VLCD recipients and 2.9% (1/34) of VLCD recipients dying at one year (*p*=0.6).

### 3.3. Association between Having Received a VLCD Graft and Hospital Length of Stay

Using a multivariate logistic regression and after adjusting to known risk factors of prolonged hospital stay, we found that the receipt of a VLCD graft was not associated with a prolonged LOS (Table 4).

### 3.4. Liver Steatosis in Recipient of a VLCD Graft

We investigated the rates of *de novo* SLD in the 26 non-SLD recipients who received a VLCD graft. Among these, 19 recipients had follow-up imaging (2/19 ultrasound, 2/19 CT scan) or biopsies (15/19) performed at the 1-year interval posttransplant. Of these, five recipients had >5% steatosis within the graft.

## 4. Discussion

Hepatic steatosis excludes potential living donors from organ donation [16]. As the prevalence of obesity and steatosis continues to rise worldwide, there is an increasing interest in developing strategies to solve this problem. In 2012, our program implemented the use of OPTIFAST VLCD in prospective living liver donors with evidence of steatosis of >7% and in those with BMIs of >30 kg/m^2^, marking us as a unique program to use this approach. We reported previously that this treatment has excellent compliance and is safe in donors [13]. The current study is the first to compare the outcomes of living donor liver transplantation recipients who received a graft from VLCD-treated donors and VLCD-untreated donors. We show that recipient outcomes are similar between the two groups, with respect to complications, ICU and hospital stay, and mortality rates.

Other approaches to treat obesity and reduce steatosis include exercise and calorie restriction; however, they require long treatment periods, which is a notable drawback as compared to OPTIFAST VLCD treatment [17, 18]. A previous study demonstrated the efficacy of a combination of a high protein diet, exercise, and bezafibrate administered for 2–8 weeks, resulting in a significant reduction in steatosis without adversely affecting recipient outcomes when compared to recipients who received a lean graft [19]. However, the sample size in that study was small (*n* = 7), which limits the generalizability of their findings. Another study reported a reduction in hepatic steatosis with omega-3 fatty acid treatment, but recipient outcomes were not reported [20]. Moreover, it should be acknowledged that novel pharmacotherapeutic interventions which promote weight loss including the glucagon-like peptide 1 (GLP-1) receptor and gastric inhibitory polypeptide (GIP) agonists hold promise in reducing steatosis of possible donors [21].

Several studies have shown that hepatic steatosis is associated with increased ischemia-reperfusion injury, as measured by the mean peak of ALT following transplantation [22, 23]. Interestingly, we found no significant difference in ALT values in patients who received a graft from VLCD-treated and VLCD-untreated donors, which supports the efficacy of VLCD in reducing steatosis.

We then examined the recurrence of liver steatosis in the 26 non-SLD recipients who received a VLCD graft, with the hypothesis that these patients might be at risk of developing steatosis as suggested by prior single-center retrospective studies [24, 25]. Among the VLCD graft recipients, five individuals were diagnosed with SLD during their one-year follow-up period. However, retrospectively investigating the development of liver steatosis is challenging due to variations in the timing of imaging and biopsies. As a result, new cases of steatosis that might have emerged later could potentially have been overlooked.

Our study has certain limitations, including its single-center design and the limited sample size of 34 patients who received a VLCD graft. Future multicenter studies should be conducted to validate our results. Moreover, the retrospective design prevents us from definitively ruling out an increased risk of hepatic steatosis after transplantation in VLCD recipients, although our one-year follow-up did not provide substantial evidence for this.

## 5. Conclusion

This study provides further evidence supporting the use of grafts from VLCD-treated donors, with comparable short-term and long-term outcomes to non-VLCD graft recipients. These findings could help to expand the pool of potential living donors.

## Figures and Tables

**Table 1:** Donor characteristics before donation.

*n* = 199	Non-VLCD donors *n* = 165	VLCD donors *n* = 34	*P* value
Sex, female, *n* (%)	102 (62.2%)	20 (58.8%)	0.4
Age at donation (years), median (IQR)	35 (27–44)	33 (29–43)	0.8
BMI at donation (kg/m^2^), median (IQR)	25.2 (22.6–27.7)	28.1 (26.5–29.7)	<0.01
AST before donation (IU/L), median (IQR)	18 (15–22)	19 (15–22)	0.7
ALT before donation (IU/L), median (IQR)	18 (15–22)	20.5 (14–25)	0.3
Total bilirubin before donation (*μ*mol/L), median (IQR)	10 (7–13)	9.5 (7–17)	0.8
INR before donation, median (IQR)	0.9 (0.9–1)	1 (1–1.1)	<0.01

The Mann–Whitney *U* test was used for continuous variables and the exact Fisher test for categorical variables.

**Table 2:** Recipient baseline characteristics.

*n* = 199	Non-VLCD recipient *n* = 165	VLCD recipient *n* = 34	*P* value
Sex, female, *n* (%)	83 (50.6%)	13 (38.2%)	0.3
Age at transplant (years), median (IQR)	55 (45–63)	59 (46–64)	0.4
*Primary diagnosis, n (%)*			0.9
Primary sclerosing cholangitis	39 (23.6%)	8 (23.5%)	
SLD	33 (20%)	8 (23.5%)	
B or C hepatitis	27 (16.4%)	7 (20.6%)	
Hepatocellular carcinoma	8 (4.9%)	0 (0%)	
Autoimmune hepatitis	13 (7.9%)	2 (5.9%)	
Alcohol liver cirrhosis	19 (11.5%)	6 (17.7%)	
Fulminant hepatitis	2 (1.2%)	0 (0%)	
Retransplant	1 (0.6%)	0 (0%)	
Others^†^	23 (13.9%)	1 (2.9%)	
*Secondary diagnosis, n (%)*			0.5
None	104 (63%)	21 (61.8%)	
Primary sclerosing cholangitis	3 (1.8%)	0 (0%)	
SLD	6 (3.6%)	0	
B or C hepatitis	2 (1.2%)	0	
Hepatocellular carcinoma	40 (24.2%)	9 (26.5%)	
Autoimmune hepatitis	2 (1.2%)	0	
Alcohol liver cirrhosis	7 (4.2%)	3 (8.8%)	
Others^†^	1 (0.6%)	1 (2.9%)	
AST before LT (IU/L), median (IQR)	53 (35–92)	52.5 (35–73)	0.6
ALT before LT (IU/L), median (IQR)	33 (20–58)	33.5 (25–67)	0.6
Total bilirubin before LT (*μ*mol/L), median (IQR)	48 (24–88)	43 (18–77)	0.5
INR before LT, median (IQR)	1.4 (1.2–1.7)	1.4 (1.2–1.6)	0.8
MELD before LT, median (IQR)	19 (14–23)	19.5 (14–22)	0.8

SLD, steatotic liver disease; AST, aspartate transaminase; ALT, alanine transaminase; MELD, model for end-stage liver disease. ^†^Including metabolic liver disease and drug-induced liver disease that are not fulminant hepatitis. The Mann–Whitney *U* test was used for continuous variables and the exact Fisher for categorical variables.

**Table 3:** Recipient outcomes.

*n* = 199	Non-VLCD recipient *n* = 165	VLCD recipient *n* = 34	*P* value
*Peritransplantation period*			
Cold ischemia time (minutes), median (IQR)	114 (76–163)	83 (56.5–152)	0.2
Warm ischemia time (minutes), median (IQR)	47.5 (39.5–61)	46.5 (41–62)	0.9
Duration of surgery (minutes), median (IQR)	557.5 (480–650)	508.5 (435–600)	0.09
Graft-to-recipient weight ratio, median (IQR)	1.1 (1–1.4)	1.3 (1–1.7)	0.04
*Post-liver transplant period*			
Peak AST post-LT (IU/L), median (IQR)	552 (300–920)	419 (238–718)	0.3
Peak ALT post-LT (IU/L), median (IQR)	624 (357–946)	525 (308–745)	0.3
Peak total bilirubin post-LT (*μ*mol/L), median (IQR)	110 (73–174)	118 (73–156)	0.9
Peak INR post-LT, median (IQR)	2.2 (1.7–2.6)	1 (1–1.25)	<0.01
Any complications^†^, *n* (%)	38 (23%)	11 (32.4%)	0.3
Biliary stricture, *n* (%)	20 (12.1%)	6 (17.7%)	0.4
Hepatic thrombosis, *n* (%)	5 (3%)	2 (5.9%)	0.3
Small-for-size syndrome, *n* (%)	1 (0.6%)	1 (2.9%)	0.3
Abdominal bleeding, *n* (%)	6 (3.6%)	0 (0%)	0.6
Need for surgical revision, *n* (%)	3 (1.8%)	0 (0)	0.6
Infection during hospital stay, *n* (%)	9 (5.5%)	2 (6.1%)	1
ICU admission, *n* (%)	117 (70.9%)	24 (70.6%)	1
ICU LOS (days), median (IQR)	2 (1–4)	2 (2–4)	0.8
Hospital LOS (days), median (IQR)	13 (9–22)	13 (9–24)	0.8
30-day mortality, *n* (%)	1 (0.6%)	1 (2.9%)	0.3
90-day mortality, *n* (%)	3 (1.8%)	1 (2.9%)	0.5
One-year mortality, *n* (%)	7 (4.2%)	1 (2.9%)	0.6

AST, aspartate transaminase; ALT, alanine transaminase; ICU, intensive care unit; LOS, length of stay. Peak values are defined as the highest laboratory value in the first 5 days following the liver transplant. ^†^Any complications are defined as follows: hepatic thrombosis, abdominal bleeding, posttransplant infection, need for surgical revision, and small-for-size syndrome. The Mann–Whitney *U* test was used for continuous variables and the exact Fisher test for categorial variables.

**Table 4:** Association between type of graft and hospital length of stay.

Hospital		
LOS >13 days	OR (95% CI)	*p* value
*n* = 198		

VLCD donor	1.1 (0.5–2.3)	0.9
*MELD score (per point)*		
<16	Ref	1
16–22	1.6 (0.8–3.2)	0.2
≥22	4 (1.9–8.4)	<0.01
Age ≥ 60 years	1.4 (0.8–2.6)	0.2

LOS, length of stay; MELD, model for end-stage liver disease. Results of the logistic regression are expressed in odds ratio (OR) with 95% confidence intervals (95% CI).

## Data Availability

The data used to support the findings of this study are available from the corresponding author upon request.

## Abbreviations

ICU: Intensive care unit
LOS: Length of stay
MELD: Model for end-stage liver disease
SLD: Steatotic liver disease
VLCD: Very low calorie diet.

## References

[1] Riazi K., Azhari H., Charette J. H. (2022). The prevalence and incidence of NAFLD worldwide: a systematic review and meta-analysis. *The Lancet Gastroenterology & Hepatology*.

[2] Rinella M. E., Alonso E., Rao S. (2001). Body mass index as a predictor of hepatic steatosis in living liver donors. *Liver Transplantation*.

[3] Alqahtani S. A., Gurakar A., Tamim H. (2022). Regional and national trends of adult living donor liver transplantation in the United States over the last two decades. *Journal Clin Transl Hepatol*.

[4] Lucidi V., Gustot T., Moreno C., Donckier V. (2015). Liver transplantation in the context of organ shortage: toward extension and restriction of indications considering recent clinical data and ethical framework. *Current Opinion in Critical Care*.

[5] Adam R., McMaster P., O[apos ]Grady J. G. (2003). Evolution of liver transplantation in europe: report of the European liver transplant registry. *Liver Transplantation*.

[6] Briceño J., Ciria R., De La Mata M., Rufián S., López-Cillero P. (2010). Prediction of graft dysfunction based on extended criteria donors in the model for end-stage liver disease score era. *Transplantation*.

[7] Adam R., Reynes M., Johann M. The outcome of steatotic grafts in liver transplantation. *Transplantation Proceedings*.

[8] Kulik U., Lehner F., Klempnauer J. B. J., Borlak J. (2017). Primary non-function is frequently associated with fatty liver allografts and high mortality after re-transplantation. *Liver International*.

[9] Noujaim H. M., De Ville De Goyet J., Montero E. F. S. (2009). Expanding postmortem donor pool using steatotic liver grafts: a new look. *Transplantation*.

[10] Linares I., Hamar M., Selzner N., Selzner M. (2019). Steatosis in liver transplantation: current limitations and future strategies. *Transplantation*.

[11] Gudzune K. A., Doshi R. S., Mehta A. K. (2015). Efficacy of commercial weight-loss programs. *Annals of Internal Medicine*.

[12] Lewis M. C., Phillips M. L., Slavotinek J. P., Kow L., Thompson C. H., Toouli J. (2006). Change in liver size and fat content after treatment with Optifast® very low calorie diet. *Obesity Surgery*.

[13] Doyle A., Adeyi O., Khalili K. (2016). Treatment with Optifast reduces hepatic steatosis and increases candidacy rates for living donor liver transplantation. *Liver Transplantation*.

[14] Narayanan P., Mara K., Izzy M. (2019). Recurrent or de Novo allograft steatosis and long-term outcomes after liver transplantation. *Transplantation*.

[15] Feng Y., Han Z., Wang X., Chen H., Li Y. (2020). Association of graft-to-recipient weight ratio with the prognosis following liver transplantation: a meta-analysis. *Journal of Gastrointestinal Surgery*.

[16] Mazilescu L. I., Selzner M., Selzner N. (2021). Defatting strategies in the current era of liver steatosis. *JHEP Reports*.

[17] Chung J. H., Ryu J. H., Yang K. H. (2020). Efficacy and safety of weight reduction of the donor in hepatic steatosis for living donor liver transplantation. *Annals of Transplantation*.

[18] Fujii Y., Kawamura N., Zaitsu M. (2020). Outcome of living-donor liver transplantation using grafts from donors treated for fatty liver. *Annals of Transplantation*.

[19] Nakamuta M., Morizono S., Soejima Y. (2005). Short-term intensive treatment for donors with hepatic steatosis in living-donor liver transplantation. *Transplantation*.

[20] Clavien P. A., Oberkofler C. E., Raptis D. A., Lehmann K., Rickenbacher A., El-Badry A. M. (2010). What is critical for liver surgery and partial liver transplantation: size or quality?. *Hepatology*.

[21] Newsome P. N., Buchholtz K., Cusi K. (2021). A placebo-controlled trial of subcutaneous semaglutide in nonalcoholic steatohepatitis. *New England Journal of Medicine*.

[22] Perez-Daga J. A., Santoyo J., Suárez M. A. (2006). Influence of degree of hepatic steatosis on graft function and postoperative complications of liver transplantation. *Transplantation Proceedings*.

[23] Selzner N., Selzner M., Jochum W., Amann-Vesti B., Graf R., Clavien P. A. (2006). Mouse livers with macrosteatosis are more susceptible to normothermic ischemic injury than those with microsteatosis. *Journal of Hepatology*.

[24] Kim H., Lee K., Lee K. W. (2014). Histologically proven non-alcoholic fatty liver disease and clinically related factors in recipients after liver transplantation. *Clinical Transplantation*.

[25] Dumortier J., Giostra E., Belbouab S. (2010). Non-alcoholic fatty liver disease in liver transplant recipients: another story of seed and soil. *American Journal of Gastroenterology*.

